# Nursing students’ views of the impact of geriatric role-play workshops on professional competencies: survey

**DOI:** 10.1186/s12912-023-01373-y

**Published:** 2023-06-14

**Authors:** Ester Benko, Melita Peršolja

**Affiliations:** 1 Faculty of Health Sciences, University of Primorska, Koper, Slovenia; 2 Faculty of Health Sciences, Unit Vipava, University of Primorska, Koper, Slovenia

**Keywords:** Geriatric nursing, Nursing students, Professional competence, Role playing

## Abstract

**Background:**

Simulation and role-playing are the most commonly used experiential teaching methods in nursing education. The purpose of the study was to describe the impact of geriatric role-play workshops on the knowledge and skills of nursing students. We set one hypothesis: Students believe that learning through experiential role-play improves their professional competencies.

**Methods:**

We conducted a descriptive quantitative study, collecting the data with a questionnaire. The study included 266 first-year nursing students who underwent 10 h of role-playing workshops in geriatric nursing in 2021. The questionnaire was compiled for the purpose of the present study, and its’ internal consistency was 0.844 (*n* = 27). We used descriptive and correlation statistical analysis.

**Results:**

Respondents were convinced that they gained and consolidated knowledge and connected theory with practice through role-playing. They especially emphasized the abilities they acquired to communicate in a group, engage in constructive reflection, be more sensitive to one’s own emotions, and feel empathy.

**Conclusions:**

Respondents understand the use of the role-play method as an effective form of learning in geriatric nursing. They are convinced that they will be able to use the experience when working with an elderly patient in a clinical setting.

## Background

In Slovenia the undergraduate study program in Nursing in line with the European directives and the Bologna process contains at least 2,300 h of clinical training in teaching institutions [1]. The nursing study program enables graduates to acquire the competencies needed for independent work. Such competencies are defined based on the degree to which each individual can use their knowledge, skills and judgment related to the implementation of professional practice [2]. Nehring and Lashley [3] note that nowadays it is expected that during their studies a graduate nurse will have acquired the competencies necessary to pursue the profession – for example, to be able to perform nursing interventions, think critically, solve problems, make clinical judgments – and develop from a novice to an experienced graduate nurse in the shortest time possible.

Today nursing higher education is facing many changes, which are the result of rapid and complex social change, advances in science and technology, greater enrolment of students in nursing study programs, and consequently the problem of providing adequate clinical training. Due to the growth in student numbers and the increasingly demanding goals of study programs, it is important to introduce modern teaching methods into the curriculum, and simulation and role-playing are among the most commonly used experiential learning methods in nursing education [4–10].

Ertmer et al. [11] define role-play as a method of experiential learning in which students play assigned roles in certain scenarios, with the aim of providing focused practice and feedback on learned skills. Role-playing differs from simulation, in that the latter precisely defines the characters’ desires and goals, while role-playing gives students more freedom to think about how to act in a given situation. DeNeve and Heppner [12] describe role-playing as creating more or less structured situations in which students’ behaviour is improvised in accordance with their notions of the roles assigned to them. The participant temporarily assumes the prescribed role, and thus the thinking, feeling and behaviour of someone else. The actor is not personally responsible for the behaviour they enact in the role, as their goal is only a good presentation or imitation of the role itself.

Experiential learning methods enable the acquisition and consolidation of knowledge and a variety of skills that prepare students for a real clinical environment in a simulated one [6, 13], thus enabling the transfer of knowledge from lecture halls and special nursing classrooms to the clinical environment [14]. In the field of medicine and nursing, for example, communication skills training plays a very important role in undergraduate, postgraduate and continuing education [15, 16]. Rowles and Brighan [17] believe that role-playing is particularly suitable for adult participants, as it is linked to everyday life, and active participation further enhances decision-making skills. According to some authors [18, 19], role-play is also often used as a teaching method in the field of ethics education, as it promotes the process of ethical decision-making.

Role-plays allows students to delve deeper into their learning [20], helping them to develop and improve the content of skills, as well as the overall skills needed for a successful future in the profession, by incorporating real-world problems [6, 21]. Role-playing is also a commonly used method for developing decision-making skills [22], leadership and listening [23], problem solving [24] and the promotion of accountability [25]. Role-playing provides an opportunity to develop many skills – such as team-work [9, 10, 26–28], improving cultural awareness [29, 30], caring for older adults [19, 31], increasing assertiveness [3, 32], self-confidence [3, 28], and reducing feelings of anxiety and stress [26] – as it provides a safe learning environment with less anxiety and fear [23, 33, 34]. Many authors note the benefits of role-playing, including increased motivation [26, 33, 35], feedback or the possibility of immediate sharing of ideas and thoughts in the reflection phase [29, 33, 36]. In addition to its efficiency in promoting reflection, some authors [21, 28] see role-playing as a very good way of linking theory and practice.

The aim of this research was to describe the correlation of role-play with the professional competences of nursing students with regard to working with older patients. We set one hypothesis: Students believe that learning through experiential role-play improves their geriatric professional competencies.

## Methods

### Sample

As the study target, the population of first year full-time students of the nursing undergraduate study program at the Faculty of the Health Sciences University of Primorska was identified. The study included the population from the study years 2016/2017 (*n* = 134), 2017/2018 (*n* = 133), and 2019/2020 (*n* = 129). In total 396 students were eligible as they participated in the role-play workshops within the obligatory subject *Nursing of the elderly*. Of the population, 67,18% (*n* = 266) were willing to complete the questionnaire. The confidence level was set at 95%, and the sample size needed for a 5% confidence interval was a minimum of 195 students.

### Setting

Role-playing workshops were included in the study program for the subject *Nursing of the elderly*, based on a pilot study [37]. The course of students’ clinical training included training in nursing homes and in special classrooms at the faculty. We organized students into groups of fourteen to sixteen. Each group was involved in 10 h of workshops on two consecutive days before the clinical training in a nursing home.

### Role-play workshop

The role-play workshops were conducted in four phases and led by a lecturer of gerontological nursing (the first author), who introduced the students to the purpose, content, goals, and course of the workshops. The role-plays were aimed toward the nursing home setting. Workshops were held in a nursing skills laboratory that simulates a room in a nursing home. In addition, the students had at their disposal an aging simulator (KOKEN Aged simulation set), food and microwave oven (to prepare breakfast), and all the devices from the nursing skills laboratory (such as a wheelchair, ambulance stretcher, positional pillows, etc.).

In the preparation phase, the lecturer distributed the scenarios with written instructions for the role of the nurse and separately for the role of the patient. The patient roles in the scenarios related to various diseases and problems of old age, such as arthritis, diabetes mellitus, disability after stroke, Parkinson's disease, vision and hearing disorders. This was followed by the student who was in the role of a patient getting ready for the role-play, which was done by the lecturer “disabling” them with an aging simulator.

Using the aging simulator, students had the opportunity to briefly put themselves in the shoes of an older patient with certain limitations. For example, a visually impaired or blind patient, a patient with Parkinson’s disease, a patient with arthroses, swallowing disorders, hearing problems, and so on. To make the experience as close as possible to the “real” one, the students were also fed by someone else, a student in the role of a nurse. They also received some medication, in the form of candies, and were transferred to a wheelchair or recumbent wheelchair and “taken to be examined” outside the classroom. The decision on the scenarios was made by the authors, based on more than 25 years of experience in working with geriatric patients.

Students in groups of two played their roles according to the prepared scenarios. Each student had the opportunity to play both the role of the patient and that of the nurse. Each scenario lasted from five to a maximum of fifteen minutes, and was followed by a debriefing. In this generalized discussion, each participant had the opportunity to express their observations, feelings, opinions, or suggestions. The lecturer encouraged students to talk with questions such as: “How did you feel as a patient?”, “Did you feel safe when you were transferred to a wheelchair?”, “How did you feel when you were blind and the nurse fed you?”, “Do you think this experience will have an impact on the way you work alongside patients in a real-life setting?”.

### Instrument

A descriptive quantitative non-experimental research method was used. The data were collected with a non-standardized questionnaire developed by Benko [37] based on a literature review specifically for this study. The questionnaire contained two demographic questions and four sets of statements (assessment of the impact of participation in role-play workshops, assessment of the goals set, and advantages and disadvantages of the role-play method) which were assessed using a Likert scale with a score from 1 to 5 (5 – the highest level of agreement).

The survey was anonymous, and the data were used exclusively for this study. The survey’s Cronbach’s alpha coefficient was 0.844 (*n* = 27).

### Analysis

The level of analysis was the student. Exploratory data analyses were performed to inspect the data and identify inconsistencies. Quantitative data analysis was performed using descriptive methods: mean (M), standard deviation (Sd), frequency (n), percentage (%), minimum (min) and maximum (max), Pearson’s correlation (r), or Spearman’s correlation (R). Correlation strengths were valued as follows: 0–0.09 not correlated, 0.1–0.3 weak, 0.31–0.6 medium, and 0.61–1 strong correlation [38]. The statistical confidence was set at the interval of 95%, with a risk level of 0.05. The data were statistically processed using SPSS 21.0 statistical software (IBM Corp., Group NY, USA).

### Ethical considerations

Institutional review board approval was obtained before the start of the study from the Faculty of Health Sciences, University of Primorska (Slovenia). The study was conducted in accordance with the Code of Ethics for Nurses and Nurse Assistants [39], as well as the Declaration of Helsinki [40]. Students’ consent was obtained on-site. They were informed that “research on workshops” was being conducted, provided with information regarding what would happen to the data collected, and offered the name and details of the responsible contact person when necessary.

## Results

The average age of participants was 20,19 years (Min. = 19; Max. = 41; Sd = 2.73; *n* = 318), 74 (20.5%) were men. Students described the role-play “workshops” as having a high level of benefits, with a total mean of 4.42 (Min. = 2; Max. 5; Sd = 0.53, *n* = 261). The highest scores were achieved with regard to acquiring and consolidating knowledge, enabling open group communication and increasing cognitive knowledge (Table 1). Students were convinced that the workshops helped them acquire, increase and consolidate knowledge, and integrate theoretical with practical knowledge. Besides the cognitive advantages of the workshops, the respondents also noted the benefits relating to open group communication, constructive reflection and increased emotional learning. The degree to which the perceived benefits of role-plays related to the respondents’ experience of nursing was not significant (*F*  =  2.104; Sig. 0.065; df = 5). Some benefits were significantly related to gender, as women were more convinced than men participants that the role play improves the skill of communicating with the patient (*r* = 0.125; Sig. 0.018; *n * =  357), improves awareness of emotions in oneself and other participants (*r*  =  0.195; Sig. 0.001; *n* = 353), is a fun way to acquire and consolidate knowledge (*r* = 0.207; Sig. 0.001; *n* = 357) and increases cognitive knowledge (*r*  =  0.132; Sig. 0.013; *n* =  357).

**Table 1:** Advantages of geriatric role-play workshops

Advantages	n	Min	Max	Mean	Sd
A fun way to acquire and consolidate knowledge	266	3.00	5.00	4.82	.398
Open group communication in the reflection phase	228	3.00		4.69	.541
Increases cognitive knowledge	266	2.00		4.67	.584
Enables the integration of theoretical and practical knowledge	265	2.00		4.64	.571
Consolidating and acquiring knowledge in a safe environment	266	2.00		4.63	.533
Reflection is constructive	265	2.00		4.56	.600
Gaining a sense of reality	266	1.00		4.54	.762
Increases emotional learning	266	2.00		4.47	.695
Gaining in self-confidence	265	1.00		4.38	.760
Increases motivation to learn	264	2.00		4.14	.846

*n* number, *Min* Minimum, *Max* Maximum, *Sd* Standard deviation

Achieving the geriatric role-play workshops’ pedagogic and secondary goals is important, and the students were convinced that realizing the goals of the role-play would improve their work with patients (*r* = 0.548; Sig. = 0.000; *n* = 135). In general (89.9%; *n* = 240) they (strongly) agreed that learning through geriatric role-play improved their professional competencies (Mean = 4.27; Sd = 0.68; Min. = 2; Max = 5; Mode = 4).

According to the students, their work with patients would improve due the following effects of geriatric role-play: increased motivation to study, consolidating knowledge and skills in a safe environment, using the theoretical and practical knowledge they have acquired, increased empathy and understanding, having a greater awareness of one’s own feelings and those of others, learning, having the opportunity to increase the sensitivity to own emotions, and controlling one’s own emotions in a safe environment (Table 2). Woman participants were more convinced that the role play.

**Table 2:** The correlation of the geriatric role-plays effects with its’ impact on working with patients

Effects of geriatric role-play	Impact of the workshop on working with patients		
	n	r	Sig
Role-play increases the ability to understand and empathize	137	.530^**^	.000
Role-play increases communication skills		.523^**^	
Role-play increases self-confidence		.472^**^	
Role-play allows you to consolidate the skill of assessing the patient’s condition		.463^**^	
Role-play increases learning motivation		.404^**^	
Role-play is an effective learning method		.397^**^	
Role-play increases sensitivity to one’s feelings		.387^**^	
Role-play enables learning and consolidation of knowledge and skills in a safe environment		.361^**^	

*n* number, *r* Correlation Coefficient, *Sig.* Significance

^**^Statistically significant

The approach to the patient is defined by four variables, namely the nurses’ skills in communication, feeding, transfer, and assessment (Table 3). The strongest correlation and therefore the greatest impact of geriatric role-play is shown in the improvement of the skills of communicating with the patient. Woman participants were more convinced that the role play will improve their skill of communicating with the patient (*r* = 0.125; Sig. = 0.02; *n* = 357).

**Table 3:** Assessing the impact of the geriatric role-play method on the approach to the patient

With role-play, I will …						Approach to the patient		
	N	Min	Max	Mean	Sd	Beta	p	_R_2
…improve my skills in communicating with the patient	266	1.00	5.00	4.451	.736	.380	0.00	.864
… improve my skills in feeding the patient				4.289	.797	.345	0.00	
… improve my skills in assessing the patient’s condition				4.398	.771	.202	0.00	
… improve my skills in patient transfer				4.443	.721	.143	0.01	

*n* number, *Min* Minimum, *Max* maximum, *Sd.* Standard deviation, *Beta* Regression coefficient, *p* Probability, *R*^*2*^ R-squared

The secondary goals – disseminating and deepening theoretical knowledge; connecting it with practical knowledge; improved study motivation; attention directly focused on the problem at hand; expressing one’s own emotions; awareness of emotions in oneself and other participants; providing a safe opportunity to apply the acquired knowledge, skills and emotional control; and awareness of the importance of good communication with the patient – all seem to be achieved, as their average scores were good (Mean = 3.94; Min. 2, Max. 5; Sd = 0.64) and most of the students (82.2%; *n* = 111) (strongly) agreed that the goals of role-play were realized (Table 4).

**Table 4:** Assessment of secondary goals in the geriatric role-play workshops

Goal	N	Min	Max	Mean	Sd
Awareness of the importance of good communication with the patient	266	3.00	5.00	4.73	.474
The learning situation represents a safe opportunity to apply the acquired knowledge, skills and emotional control	266	2.00		4.59	.633
Disseminating and deepening theoretical knowledge and connecting it with practical knowledge	266	1.00		4.46	.661
During the role-play, attention is directly focused on the problem at hand	264	1.00		4.44	.678
Awareness of emotions in oneself and other participants	263	2.00		4.39	.706
Expressing one’s own emotions	266	1.00		4.28	.788
Improved study motivation	266	1.00		4.04	.874

*n* number, *Min* Minimum, *Max* Maximum, *Sd.* Standard deviation

An analysis of the predictors of the dependent variable in this study – the realization of the role-play’s goals – showed that it was 73.6% predicted by improved study motivation (β = 0.467; Sig. = 0.00), the possibility of expressing one’s own emotions (β = 0.332, Sig. = 0.00), and an improved awareness of emotions in oneself and other participants (β = 0.221; Sig. = 0.002). These results show that the students’ achievements are centred on expressing and understanding emotions.

## Discussion

The results show the students believe that learning gerontological nursing through experiential role-play is an effective learning method that improves their professional competencies.

A sensory activity, like playing a completely dependent blind person while wearing an age simulation suit, allows students to put themselves in the shoes of older patients [4, 41]. In this way, they can develop more empathetic attitudes and their sensitivity to the feelings of others increases [42]. Students who have empathetic attitudes towards older patients have a better understanding of the changes and illnesses that occur in old age, and have a more positive attitude towards older people, which results in better quality care [43]. However, it should be noted that Lee and The’s [44] study of pharmacy students showed that the use of an age simulation suit is not guaranteed to increase self-rated empathy.

By experiencing or observing different situations of communication between a nurse and an older patient, students had the opportunity to improve their communication skills. Ronning and Bjorkly [21] and Cortés-Rodríguez [45] also found that role-plays could improve interprofessional communication skills among nursing students working in geriatric care. Communication skills improve as one becomes better at expressing and understanding one’s own and others’ emotions, thus becoming more confident and overcoming any fears about communicating with older adults [13]. The curriculum provides students with knowledge about the changes and problems that affect communication with older patients, due to hearing, visual, neurological and cognitive impairments. But in role-playing workshops students have the opportunity to consolidate their knowledge, which enables them to effectively communicate in similar situations in real-life.

The students who took part in this study’s survey recognized role-playing as a fun way to learn. They felt that it ensures a safe environment, where they can make mistakes without consequences. The course of the role-play varied from one group of students to another, despite the use of the same scenarios. Some students easily got into their roles and performed like real actors, others were a bit shy at the beginning, but then quickly got into it because of the relaxed atmosphere in the group, with much happy laughter. The students thus had the opportunity to gain experience and knowledge in a relaxed and fun way. The finding is in line with Pilnick et al. [46], who described role-play workshops as a safe and supportive environment for learning.

Students also had the opportunity to give feedback in a reflection phase, to express their feelings and opinions on what was done well and what could have been done better or differently in a similar situation. The role of the teacher in the reflection phase is to encourage students to think critically, as here they have the opportunity to relate specific role-play situations to theoretical knowledge and practical examples [9, 13, 47]. In this way, role-play enables the integration of theoretical and practical knowledge, and increases cognitive knowledge, and confidence. Some authors [22, 48, 49] have also reported improved decision-making and problem-solving skills after role-play workshops, while Dorri et al. [6] noted that role-play learning also improves self-efficacy and motivation to learn.

One limitation of this study is that all the participants were full-time first-year students, and in a future work it would also be useful to include part-time students. Another limitation is the content and the number of scenarios which were used with the role-plays. These were limited to situations or nursing interventions that students deal with in the first year of the curriculum, and that they perform on a daily basis in the clinical setting, and in the future a broader range of situations could be used. Here it should be noted that the number of scenarios used in the role-plays was limited by the timeframe in which the workshops took place – two days of five school hours for each group of students. Future research on geriatric role-play in nursing should be a follow-up study, which will allow recognition of the impact of the activity on educational goals.

## Conclusions

Geriatric role-playing is an innovative pedagogical method, which allows students to prepare for the real clinical environment in simulated conditions. This sensory activity improves students’ empathic attitudes towards older adults – students better understand their health issues, become more confident, and overcome fears about communicating with such patients. Combined with theoretical knowledge and clinical experience, role-playing can be part of a high-quality education program that encourages active participation in the learning process, motivates students to learn and encourages the expression and understanding of emotions.

## Data Availability

The datasets used and analysed during the current study available from the corresponding author on reasonable request.
